# Identification of Novel Targets for miR-29a Using miRNA Proteomics

**DOI:** 10.1371/journal.pone.0043243

**Published:** 2012-08-27

**Authors:** Rhishikesh Bargaje, Shivani Gupta, Ali Sarkeshik, Robin Park, Tao Xu, Maharnob Sarkar, Mahantappa Halimani, Soumya Sinha Roy, John Yates, Beena Pillai

**Affiliations:** 1 Functional Genomics Unit, Council of Scientific Industrial Research - Institute of Genomics and Integrative Biology, Delhi, India; 2 Department of Chemical Physiology, The Scripps Research Institute, La Jolla, California, United States of America; IRCCS-Policlinico San Donato, Italy

## Abstract

MicroRNAs (miRNAs) are short regulatory RNA molecules that interfere with the expression of target mRNA by binding to complementary sequences. Currently, the most common method for identification of targets of miRNAs is computational prediction based on free energy change calculations, target site accessibility and conservation. Such algorithms predict hundreds of targets for each miRNA, necessitating tedious experimentation to identify the few functional targets. Here we explore the utility of miRNA-proteomics as an approach to identifying functional miRNA targets. We used Stable Isotope Labeling by amino acids in cell culture (SILAC) based proteomics to detect differences in protein expression induced by the over-expression of miR-34a and miR-29a. Over-expression of miR-29a, a miRNA expressed in the brain and in cells of the blood lineage, resulted in the differential expression of a set of proteins. Gene Ontology based classification showed that a significant sub-set of these targets, including Voltage Dependent Anion Channel 1 and 2 (VDAC1 and VDAC2) and ATP synthetase, were mitochondrial proteins involved in apoptosis. Using reporter assays, we established that miR-29a targets the 3′ Untranslated Regions (3′ UTR) of VDAC1 and VDAC2. However, due to the limited number of proteins identified using this approach and the inability to differentiate between primary and secondary effects we conclude that miRNA-proteomics is of limited utility as a high-throughput alternative for sensitive and unbiased miRNA target identification. However, this approach was valuable for rapid assessment of the impact of the miRNAs on the cellular proteome and its biological role in apoptosis.

## Introduction

miRNAs are small regulatory RNA molecules produced by almost all eukaryotic cells, by enzymatic processing of stem loop precursor RNA molecules [1], [2]. The mature miRNAs, of 19–21 nt, bind to partially complementary target regions in messenger RNA molecules, typically in the 3′UTR, in association with proteins of the miRNP complex [1], [2]. The binding of miRNA to the target mRNA can result in target cleavage, destabilization through deadenylation of the target [3], [4] and translational inhibition [5], [6]. Barring a few exceptions, miRNA binding is known to down-regulate the expression of protein from the target. Each miRNA can potentially bind to hundreds of targets in the cell [7]. Each target transcript can harbor several different miRNA binding sites resulting in a complex miRNA-target interaction network that regulates translation of eukaryotic genes and consequently, the proteomic profile of cells.

The differential expression of miRNAs in cancer, their ability to modulate apoptosis and the known interaction of miRNAs with oncogenes and tumor suppressors, taken together, suggest a significant role for miRNAs in cancer and apoptosis [8]–[11]. Although identification of differentially expressed miRNAs in cancers is supported by rapid high-throughput methodologies [12], the identification of their targets is by and large done through experimental validation of individual miRNA-target pairs. The targets of miRNAs can be predicted by computational methods that look for complementarity to the miRNA sequence and a low free energy of binding that favors miRNA-target interaction [13]. miRNA-target interaction is usually validated through reporter assays wherein the activity of a reporter gene expressed in fusion to the 3′UTR being tested is monitored in cells that ectopically express the miRNA. miRNAs can impact stability and translation of the target mRNA eventually resulting in a net reduction in the steady state level of the protein product of the target. Therefore, several groups have tried to identify targets of miRNAs, by microarray based profiling of messenger RNA in cells over-expressing the miRNA [14]. Another approach to high-throughput identification of miRNA targets is to immunoprecipitate the miRNA-target complex using antibodies against the protein components of the miRNP machinery [15], [16]. The co-immunoprecipitated miRNAs and targets can then be detected by hybridization to microarrays or sequencing. Finally computational prediction of targets is used in conjunction with the immunoprecipitation data to identify miRNA-target pairs. This approach, encompassing methods like High-throughput sequencing of RNA isolated by crosslinking immunoprecipitation (HITS-CLIP) [17] and Photoactivatable-Ribonucleoside-Enhanced Crosslinking and Immunoprecipitation (PAR-CLIP) [18] is dependent on the quality and stringency of the immuno-precipitation and requires several time-consuming steps and standardization of antibody concentration to minimize non-specific detection of targets. Most recently, polysome profiling of mRNA species after over-expression of three different miRNA has been used to capture functional mRNA targets by virtue of their reduced occurrence in actively translating poly-ribosomal fractions [19]. Irrespective of the mode of action of miRNA, through translational repression or active degradation and destabilization of RNAs, the target protein levels are expected to be reduced. Hence, proteomics can also be used to identify the role of miRNA-target interaction. This approach has been used with proteomics methods like SILAC [20]–[24], pulsed SILAC [25] and Isobaric tags for relative and absolute quantitation (iTRAQ) [26] to identify the targets of miRNAs and their effect on the overall proteomic profile of cells. Here we used SILAC to study the effect of two apoptosis related miRNAs, miR-34a and miR-29a.

miR-34a is a highly conserved pro-apoptotic miRNA that inhibits cell proliferation in normal cells and is induced by p53 in response to DNA damage [11]. Loss of miR-34a has been shown in several cancers including neuroblastoma [27], pancreatic cancer [28] and non-small-cell lung cancer [29]. Some of the direct targets of miR-34a that allow it to inhibit cell proliferation include BCL2 [29] and CDK4 [30]. Ectopic expression of miR-34a is also known to induce cellular senescence in human fibroblasts [30] and apoptosis [27], [28], [31], [32]. miR-29a has been repeatedly implicated in apoptosis, but its pro and anti-apoptotic functions have not been resolved [34], [35], [36], [37], [38]. The known targets of miR-29a include members of the Bax family of anti-apoptotic proteins [33] and p85α [34]. The ability of miR-29a to repress CDC42 and p85α results in activation of p53 and induces apoptosis [34]. miR-29a is down regulated in cancers, like hepatocellular carcinoma and acute myeloid leukemia [35], [36] and during neuronal cell death. miR-29a can target BACE1, the gene that encodes beta-secretase, aberrant expression of which is implicated in Alzheimer’s disease [37]. Thus both miR-34a and miR-29a are miRNAs involved in the regulation of apoptosis. They interact closely with the p53 pathway to establish the balance between cell proliferation and cell death. Both miRNAs are also involved in differentiation of neuronal cells and their aberrant expression results in cancers. Here, we used miRNA proteomics to study the effects of over-expression and knock down of these miRNAs in Human Embryonic Kidney 293T (HEK293T) cells.

Our results show that both miRNAs have modest effects on expression of their target genes at the protein level. We compared miRNA proteomics to computational prediction of miRNA-targets and assessed their relative advantages and disadvantages. Targets of both miRNAs showed enrichment of genes in functional classes related to their cellular role, i.e. regulation of apoptosis. Using the proteomic profiling of cells over-expressing the miRNA, we show that VDAC1 and VDAC2 are novel targets of miR-29a. VDAC1 is directly involved in apoptosis and harbours a single miR-29a target site. We show that miR-29a overexpression results in downregulation of VDAC1 and VDAC2 protein. Using reporter assays, we established that miR-29a can directly target the 3′UTR of VDAC1, VDAC2 and VDAC3. In summary, using miRNA proteomics, we not only studied the effect of two apoptosis related miRNAs but also show that the mitochondrial outer membrane porin component, VDAC can be targeted by miR-29a.

## Results

### Modulation of miRNA Levels

**Figure 1:**
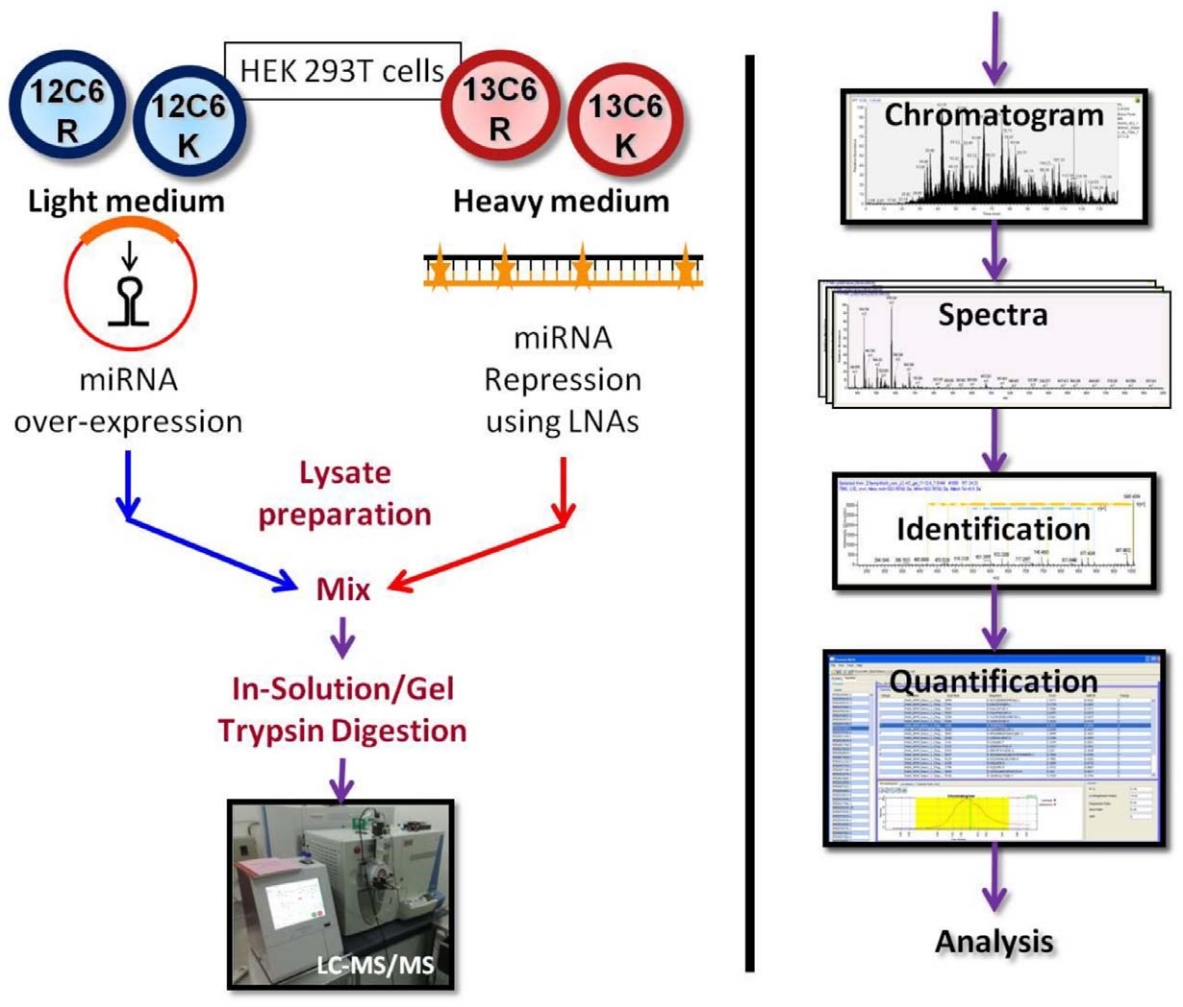
Experimental schema for miRNA-proteomics. Cells were transfected with either miRNA expressing vector or LNA modified oligonucleotide against miRNA. The lysate mix was analyzed on LTQ-ORBITRAP mass spectrometer. The data was analyzed as mentioned in materials and methods. The quantification data was then compared with other miRNA-target identification methods.

In order to study the effect of miR-34a and miR-29a, we first established conditions for ectopic over-expression and knockdown of both miRNAs. We cloned a pre-miRNA sequence along with flanking regions in appropriate vectors as mentioned in methods, and transiently transfected it into HEK293T cells. We have also used locked nucleic acid (LNA) modified antisense oligonucleotides to knock down the expression of both miRNAs (Fig. 1). HEK293T cells have detectable levels of both miR-34a and miR-29a. We initially carried out the proteomics analyses using four conditions: The miRNA overexpression was compared to cells transfected with the corresponding vector and the knockdown using LNA was compared to treatment with a mock LNA sequence. We confirmed that the cellular level of the miRNAs were increased following over-expression and decreased after treatment with LNA (Figure S1). However, the magnitude of changes in expression of proteins in each of these cases was very small. Therefore, we reasoned that the antisense based knockdown of the miRNA would help in clearing the endogenous miRNA and further enhancing the difference in expression level of the miRNA between cells transfected with the over-expression construct and anti-sense oligonucleotides.

### miRNA-proteomics

**Figure 2:**
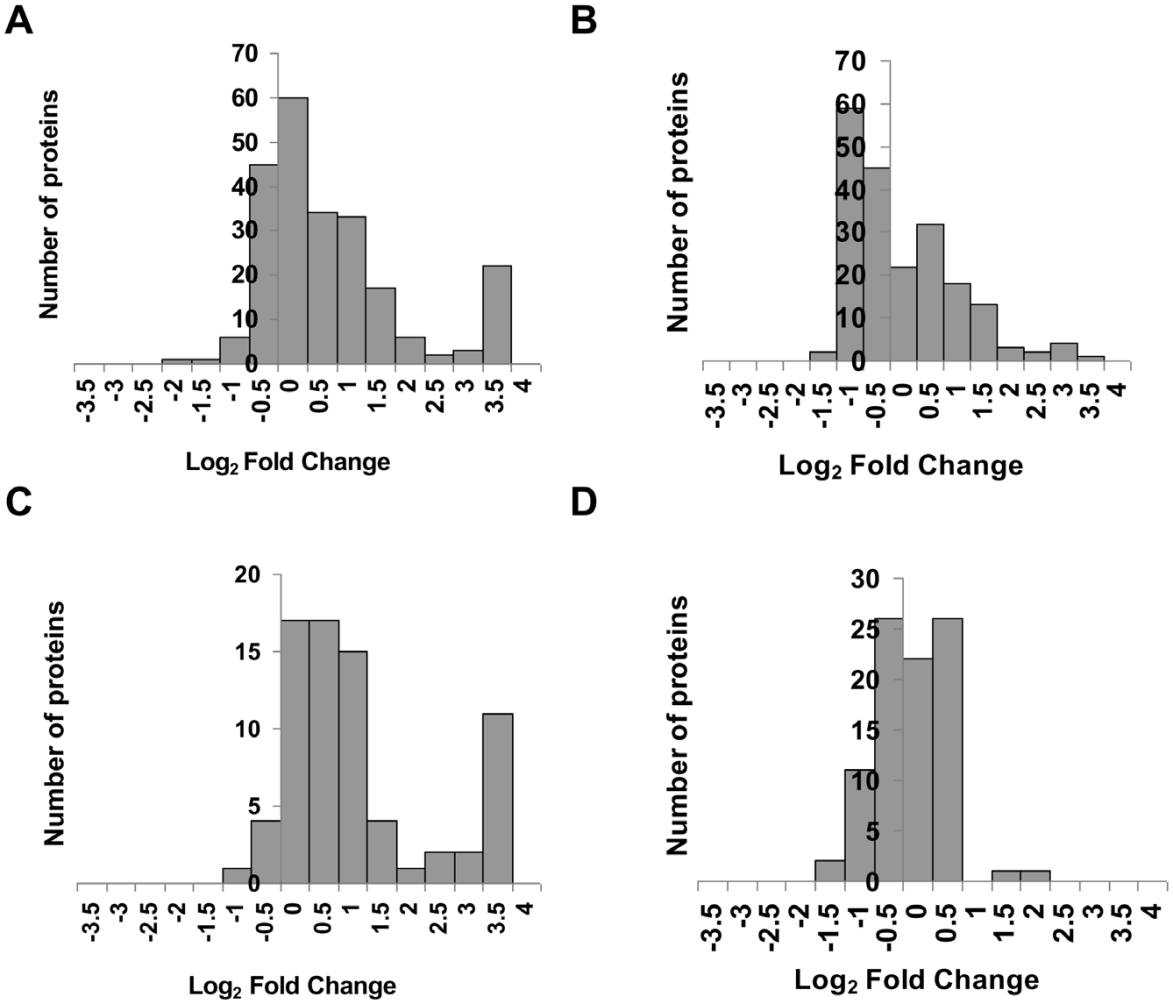
Histograms of differentially expressed proteins. Histograms show the distribution of fold changes of differentially expressed proteins in cells with altered expression of miR-34a (**A, B**) and miR-29a (**C, D**). Fold change was calculated by dividing expression level in miRNA overexpression sample by LNA transfected sample. Proteins were binned into groups based on log_2_ Fold change and plotted separately depending on p-values for differential expression (p-value <0.01: A, C; p-value <0.001: B, D).

SILAC based detection of the proteins was carried out in the transfected cells with either the over-expression construct or the LNA against the miRNA. We successfully detected 5688 proteins, in the miR-34a experiment at 1% FDR at protein level and at least two peptides per protein. These accounted for 4285 non-redundant proteins. The entire list of proteins identified and quantified in our study can be found in Table S1. In the miR-34a experiment, fold change in expression level of the protein was computed as the ratio of the peptide in miR-34a over-expression sample (labeled with the heavy isotope) to the corresponding peak in the sample wherein miR-34a was knocked down using LNA (light). On comparison of cells over-expressing miR-34a to cells where the endogenous miR-34a was cleared by anti-sense treatment, we found 230 proteins with p-value <0.01 (Fig. 2A) and 75 proteins with p-value <0.001 (Fig. 2B). For the 230 statistically significant proteins, fold changes ranged from 0.1 to 4 with 117 proteins showing upregulation while 113 proteins showing downregulation (Table 1). Amongst these 50 proteins were upregulated more than 2 fold while only 7 proteins showed more than 2 fold downregulation.

**Table 1:** Number of proteins differentially expressed after hsa-miR-34a and hsa-miR-29a over expression, with p-value <0.01 and p-value<0.001.

	No. of Proteins	p-value <0.01	p-value<0.001
**hsa-miR-34a (Total detected - 5688)**	**Differentially expressed**	230	75
	**Up-regulated**	117	23
	**Down-regulated**	113	52
**hsa-miR-29a (Total detected - 5074)**	**Differentially expressed**	201	89
	**Up-regulated**	73	28
	**Down-regulated**	128	61

Although 5074 proteins were detected in total in the miR-29a experiment, only 4527 were non-redundant hits using similar quality control as miR-34a. The entire list of proteins obtained in miR-29a study is mentioned in Table S2. On comparison of cells over-expressing miR-29a to cells where the endogenous miR-29a was cleared by anti-sense treatment, we found 201 proteins with p-value <0.01(Fig. 2C) and 89 proteins with p-value <0.001 (Fig. 2D). Out of these 201 proteins, 48 proteins were downregulated more than 2 fold while only 23 proteins showed more than 2 fold upregulation (Table 1). Overall, miR-29a resulted in a larger impact on the proteome, with 63% of the detected proteins (128 out of 201) being downregulated.

We next compared the list of differentially expressed proteins from our proteomics study to their respective list of computationally predicted targets of both miRNAs. Computational prediction of targets against miRNAs typically leads to hundreds of predicted targets and is widely held to be susceptible to false positive prediction. We used consensus prediction of targets, considering only targets commonly predicted through at least five prediction programs including TargetScan to generate a list of targets for miR-34a and miR-29a and finally checked for the presence of these targets in our list of differentially expressed proteins identified by proteomics. The consensus target prediction approach led to 213 targets for miR-34a and 350 targets for miR-29a respectively (Table S3&S4). Since number of differentially expressed proteins in miRNA-proteomics experiments is typically much lower than the total number of predicted targets, we looked for common proteins detected in our experiment and predicted as targets. In the proteomics study for miR-34a, 23 out of 213 predicted targets were detected. Similarly, for miR-29a, 40 out of 350 predicted targets were detected. A known target of miR-29a, Isoform 2 of Cell division control protein 42 homolog (CDC42) was downregulated by 1.5 fold which could not be included in our list of differentially expressed proteins since it did not clear the p value cut off.

### Functional Analysis of Differentially Expressed Proteins

The changes in the proteome can provide valuable insights about the state of the cell following miRNA over-expression and shed light on the overall effect of the miRNA. To understand the cellular role of these miRNAs as revealed through proteomics, we carried out functional classification of the differentially expressed proteins using Database for Annotation, Visualization, and Integrated Discovery (DAVID) tool. Gene Ontology (GO) classification of miR-34a up regulated targets revealed the occurrence of the GO terms, “Nucleosome assembly” and “RNA metabolic process” and related terms to be over-represented (Table 2). Although this miRNA has been implicated in regulation of cell-cycle, the differentially expressed proteins did not contain well-known cell-cycle regulators perhaps due to their low abundance. GO terms linked to mitochondrial activity were over-represented in the downregulated protein target list of miR-34a (Table 2) and miR-29a (Table 3). Taken together, GO analyses of miR-29a and miR-34a suggest that these miRNAs have overlapping effects.

**Table 2:** Gene Ontology(GO) classification of miR-34a Upregulated and Downregulated Targets.

GO Term	Criteria	No. of Genes	Gene Symbol	Differential Expression
RNA metabolism(p_corr_ value – 3.4E-10)	Biological process	24	DDX1, DDX47, DHX15, FBL, HEATR1, HNRNPA0,HNRNPA2B1, HNRNPC, HNRNPF, HNRNPH1, HNRNPM,HNRPDL, NHP2L1, NONO, NOP56, PARP1, PPAN, PPAN-P2RY11, PPP1R8, RARS, RBMX, RSL1D1, SFPQ, SRSF1,TFAM	Upregulation
Nucleosome Assembly(p_corr_ value – 1.3E-27)	Biological process	15	HIST1H1C, HIST1H1E, HIST1H2AC, HIST1H2AJ,HIST1H2BB, HIST1H2BD, HIST1H2BH, HIST1H2BJ,HIST1H2BK, HIST1H2BL, HIST1H2BM, HIST1H2BO,HIST2H2AB, HIST2H2BE, HIST2H2BF, HIST3H2BB	Upregulation
Mitochondria(p_corr_ value – 5.8E-17)	Cellular component	30	AKAP1, ACADVL, ACO2, AK2, AKAP1, ALDH18A1,ATP5A1, C21ORF33, CLTC, COX4I1, COX6C, COX7A2,CPT2, ETFB, GSTK1, HADHA, HSD17B4, MTCH2, MT-CO2,MTX1, NADKD1, NDUFAF2, NDUFS6, PHB, PNPT1,TOMM70A, TRAP1, UQCRFS1, UQCRQ, USMG5, VDAC1	Downregulation

**Table 3:** Gene Ontology (GO) classification of miR-29a Upregulated and Downregulated Targets.

GO Term	Criteria	No. of Genes	Gene Symbol	Differential Expression
RNA processing(p_corr_ value – 3.4E-2)	Biological process	8	DDX17, DKC1, EIF4A3, HNRNPM, RPL14, RPL7,SNRPD1, SRSF3	Upregulation
Nucleosome Assembly(p_corr_ value – 1.9E-5)	Biological process	4	HIST1H1E, HIST1H2BD, HIST1H2BK, HIST2H2AB	Upregulation
Mitochondria(p_corr_ value – 1.4E-4)	Cellular component	15	ATP5F1, ATP5G1, ATP5G2, ATP5G3, BSG, CCDC56, CHCHD3, HK1, MT-ATP6, PHB2, PRDX3, SLC25A12, UQCR10, VDAC1, VDAC2	Downregulation

### miR-29a Targets Voltage Dependent Anion Channel

**Figure 3:**
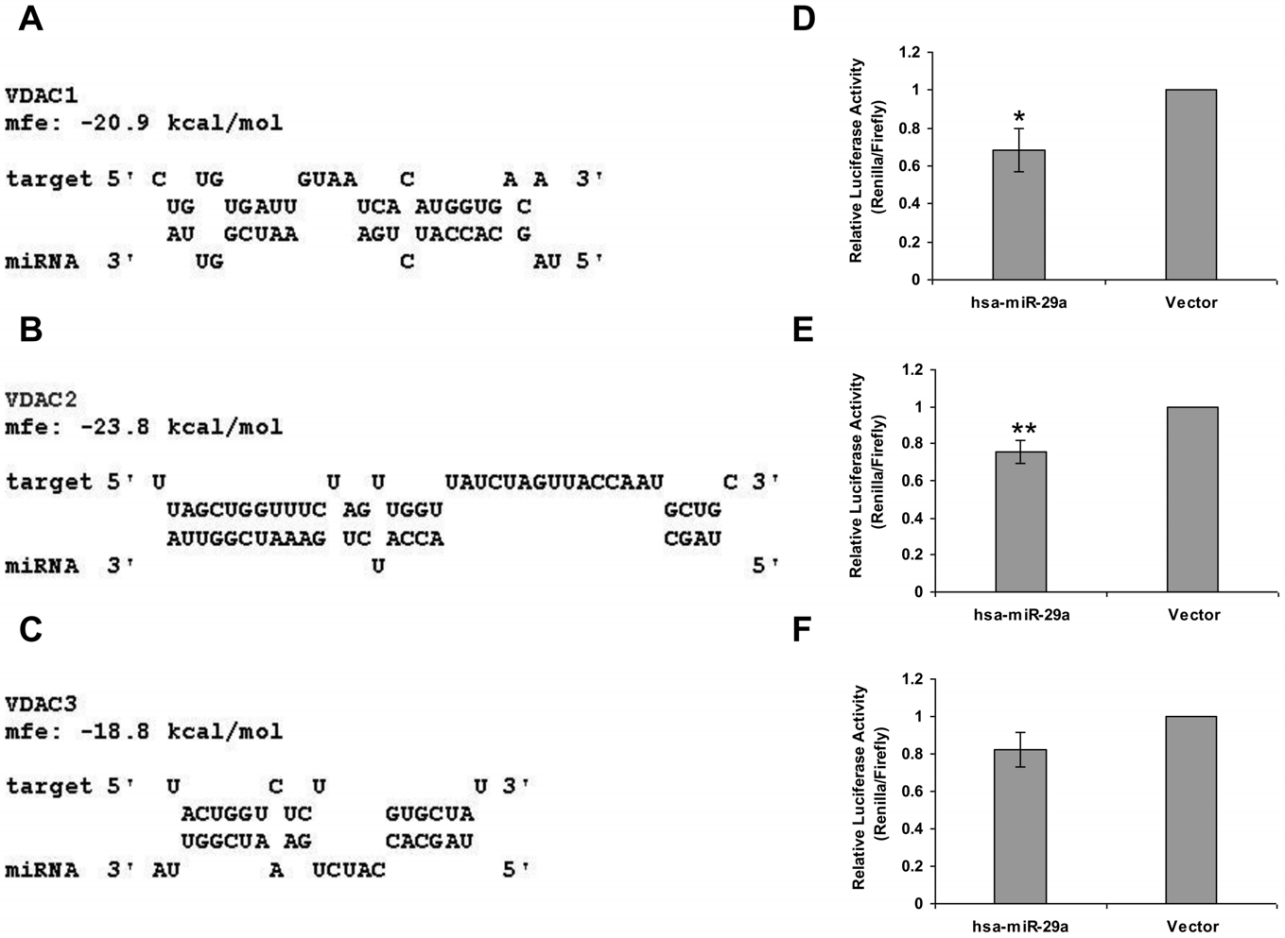
miR-29a targets 3′ untranslated region of VDAC1, 2 and 3. Binding pattern of miR-29a with the 3′UTR of the VDAC1(A),VDAC2 (B) AND VDAC3 (C). Free energies of the interactions are mentioned in Kcal/mol. (D,E,F) Luciferase activity for VDAC1, VDAC2 and VDAC3 was determined 24hours after co-transfection with Luciferase-VDAC-3′ UTR fusion and hsa-miR-29a overexpression constructs. Relative luciferase activity is shown after normalization to Firefly Luciferase. Error bars represent standard deviation from three independent experiments.

**Figure 4:**
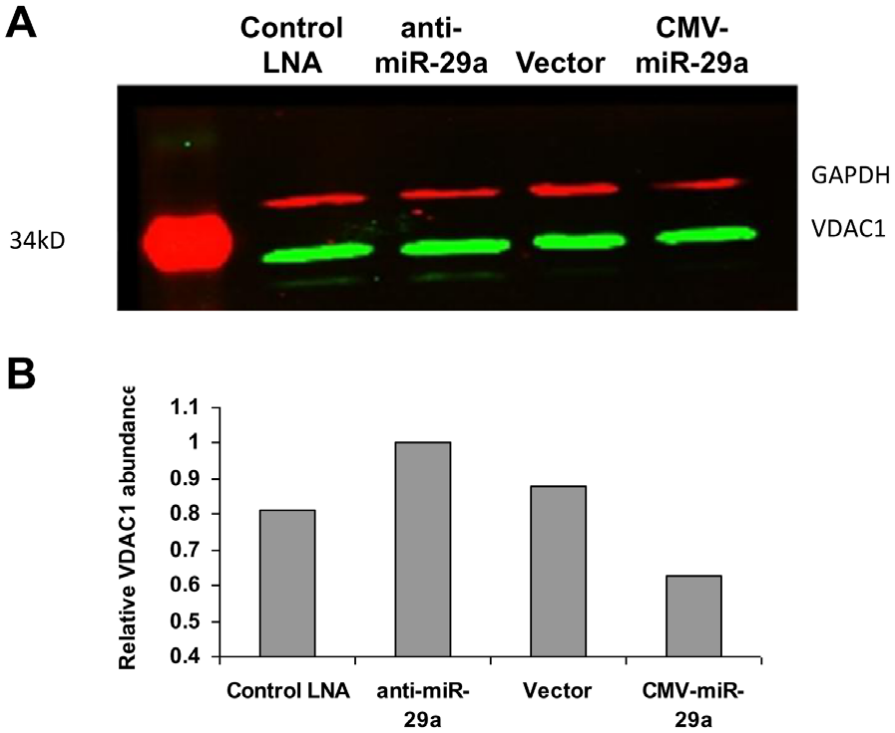
Differential Expression of VDAC1 by miR-29a. HEK293T cells were transfected with mock LNA, LNA modified anti-miR-29a, Vector or plasmid over-expressing miR-29a. (A) Immunoblotting was performed as mentioned in materials and methods and B) Band intensities quantified and normalized to loading control (GAPDH).

The differentially expressed proteins for miR-29a study included the ion channel protein VDAC1 and VDAC2. VDACs are a family of pore-forming, voltage-dependent, anion-selective channel proteins mainly located in the mitochondrial outer membrane. The VDAC (voltage-dependent anion channel) plays a central role in apoptosis, participating in the release of apoptogenic factors including cytochrome *c* into the cytoplasm [38], [39]. In mammals, three isoforms of VDAC- VDAC1, 2 and 3- exist [40]. Computational analysis done by miRNA prediction tool RNA Hybrid showed that miR-29a potentially targets 3′ UTR of VDAC 1,2 and 3 (Fig. 3A, B, C). Next, we established that the effect of miR-29a on VDAC expression was mediated through direct targeting of its 3′UTR by reporter assay. We cloned the 3′UTR of Vdac1, 2 and 3 under the Renilla luciferase reporter. Using a dual luciferase reporter construct which provides the firefly luciferase as an untargeted control, we found a 30% reduction in the luciferase activity of VDAC1/2 3′UTR fusion constructs, following miR-29a overexpression (Fig. 3D and 3E), while VDAC3 showed only a mild reduction in the luciferase activity under similar conditions (Fig. 3F). We also showed downregulation of VDAC1 at the protein level after over-expression of miR-29a by western blotting (Fig. 4). It is well known that the vast majority of miRNAs cause only mild reduction in target protein levels through single target sites. In agreement with this view, we found a 30% reduction of the target protein level when the miRNA was over-expressed. As explained before, clearing the endogenous miRNA resulted in a more pronounced effect of the miRNA, resulting in about 60% reduction of the target protein.

## Discussion

Currently, presence of hundreds of miRNAs in each eukaryotic organism is predicted or established through high-throughput experiments [41]. Each of the miRNAs can theoretically bind to hundreds of targets, as predicted by algorithms that rely on the partial complementarity between 3′UTR and miRNA sequences [7]. More refined algorithms try to add further accuracy to the predictions by imposing penalties for lack of conservation and occurrence in highly structured regions that may mask access to the site. Another approach to trim the false positives in predicted targets has been to consider only consensus targets predicted by multiple programs [42]. In spite of these approaches, the numbers of predicted targets remain well above the numbers that can be validated by reporter assays leaving the discovery of miRNA targets a tedious and slow process. Here we explore the relevance of miRNA-proteomics in identifying direct targets and indirect effects of miRNA over-expression in cultured cells, by using two miRNAs, miR-29a and miR-34a.

**Figure 5:**
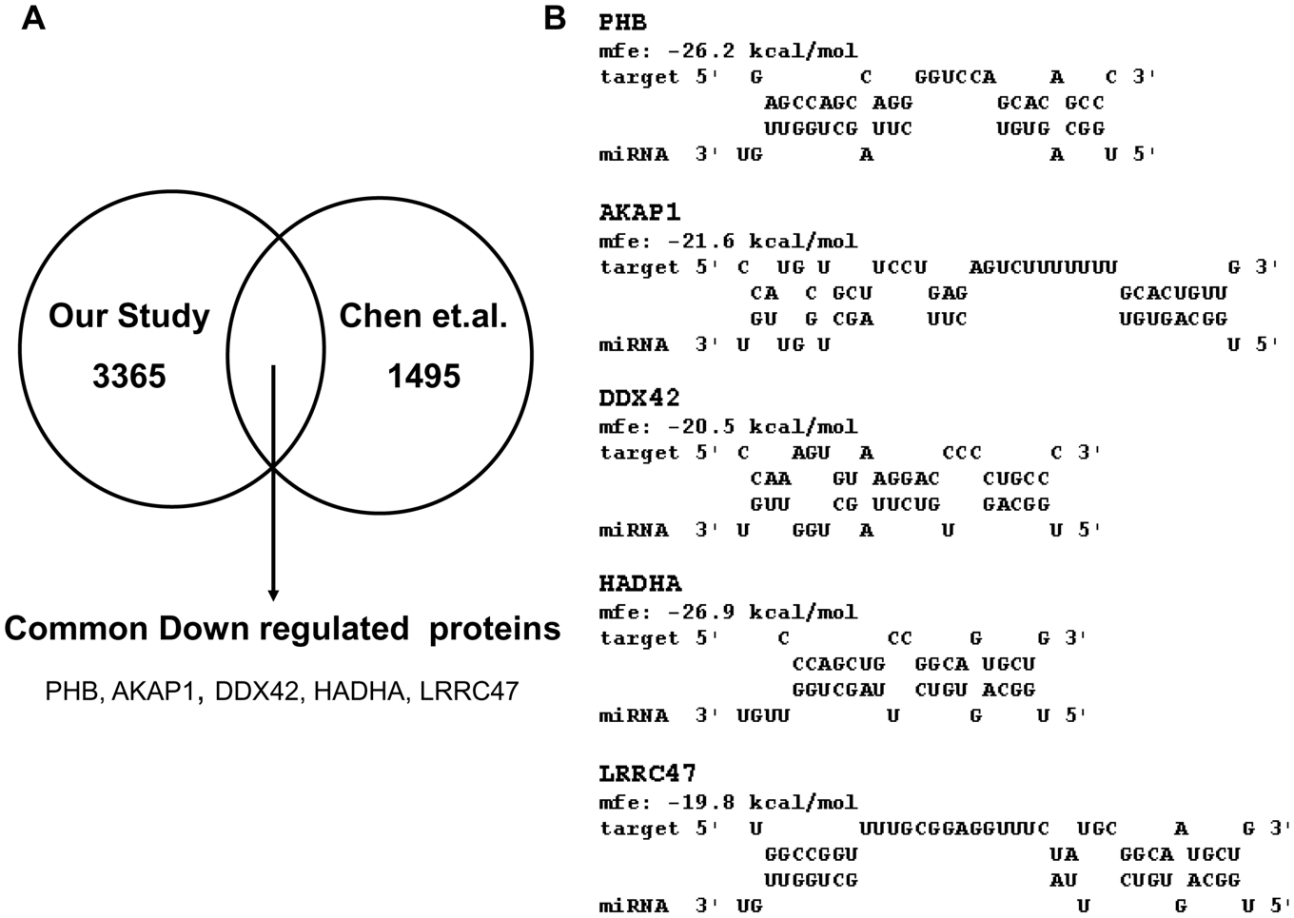
Comparative analysis of proteome profiles following miR-34 expression from two studies. Differentially expressed proteins from our study were compared to Chen et. al., 2011. A.) Numbers in the circles depict number of proteins detected in each experiment. Commonly downregulated proteins in both the studies are mentioned B.) Binding pattern of miR-34a with the 3′UTR of the commonly downregulated proteins. Free energies of the interactions are mentioned in Kcal/mol.

miR-34a is known to be induced by p53 in response to DNA damage and target anti-apoptotic proteins, thus mediating a pro-apoptotic effect. It has been also implicated in regulation of the Notch-DLL pathway during fly development and regulation of SIRT1 in human cells [43]. It is implicated in cancers due to its down-regulation in hepato-cellular carcinoma and other cancers [44], suggesting a normal pro-apoptotic role that mediates cellular senescence during development and malignancy [28]. miR-29a, on the other hand, is known to induce p53 activation by targeting CDC42, and also exert anti-apoptotic effects during brain development by keeping the expression of anti-apoptotic proteins in check [34]. Recently, two other groups [21], [45] have explored the effect of miR-34 overexpression on the proteomic profile of cells. Since the cell lines and the methodologies used are different in each study, the data collected here cannot be quantitatively compared with other experiments. We looked qualitatively for overlapping differentially expressed proteins. We found that HADHA, DDX42, LRRC47, PHB and AKAP1 proteins were commonly down regulated in both our study and Chen et.al (Fig. 5A) [21], acting as independent validation of our miR-34a-proteomics profile. All these 5 proteins also harbor potential target sites for miR-34a (Fig. 5B). Interestingly PHB, the prohibitin gene implicated in cellular senescence, development and tumour suppression, was recently predicted as a target of hsa-miR-34b using a specialized, expression based target prediction method [46].

We identified VDAC1 as a putative target of miR-29a since it harbours target sites for miR-29a and was amongst the apoptosis related proteins found to be down-regulated in our miR-29a- proteomics experiments. We established that VDAC1 and VDAC2 were targets of miR-29a. VDAC1 is known to localize to the mitochondrial outer membrane and form channels that are necessary for the release of cytochrome C into the cytoplasm. Interestingly, miR-29a is known to target several components of the mitochondrial outer membrane including MCL1, PUMA and other members of the Bcl2 family of proteins. Our study adds VDAC1 to this list of miR-29a apoptosis targets, thus suggesting a coordinated regulation of these proteins by miR-29a during apoptosis.

Besides searching for direct targets of miRNAs, miRNA-proteomics provides an opportunity to compare the cellular effect of miRNAs in terms of the differentially expressed proteins. miR-29a/b and miR-34a/b, are both known to influence apoptosis and are closely linked to p53 [11], [34]. Several groups have established that the normal role of miR-34a is to trigger apoptosis, in response to transcriptional activation by p53 [31]. In keeping with this view, expression of miR-34a is reduced in several cancers including hepatocellular carcinoma [44] and ovarian cancer [47]. miR-29a has been implicated in cancers both as an oncomiR and tumor suppressor [35], [48]. Its reported ability to target DNMT1 and 3, resulting in global hypo-methylation and upregulation of tumor suppressor genes supports a pro-apoptotic role in lung cancer cells [49]. Further, miR-29a can induce the activity of p53 by targeting CDC42 and p85alpha [34]. On the other hand its reduced expression in several myeloid cancers and its ability to cause AML in a mouse model suggests an anti-apoptotic role [35], [48]. A reduced expression of miR-29a is also associated with cell death in neuronal cells [33], [50]. To reconcile these findings, we examined the effect of both miRNAs in the same cellular context, by comparing the proteome of cells with over-expressed miRNA and reduced endogenous miRNA.

As shown in Table 2 and 3, proteins involved in nucleosome organization, primarily histones and RNA metabolism were overrepresented in the upregulated genes while, mitochondrial proteins were over-represented in down regulated genes. On the basis of the proteomic profile analyses, we propose that miR-34a and miR-29a have no apparent overlap in direct targets, but eventually result in similar effects on the whole proteome.

A few other studies have previously tried to use proteomics to identify the effects of miRNAs. Vinther et. al. first studied the effect of miR-1 [22] in HeLa cells using SILAC technology. Subsequently, SILAC was applied to capture the effects of miR-124, miR-1 and miR-181 in HeLa cells and miR-223 in knock-out mice neutrophils [20]. Pulsed SILAC was used to study the effect of miR-1, miR-155, miR-16, miR-30a and let-7b in HeLa cells [25]. The effect of miR-21 and miR-143 was also studied by miRNA-proteomics using iTRAQ [26] and SILAC [24] respectively. Selected reaction monitoring (SRM), a mass spectrometry based method has been used to validate computationally predicted targets in C. elegans [51]. Each of the previous reports independently concluded that the miRNAs showed modest effects both in number of differentially expressed proteins and fold changes in target expression. In this study we used two relatively well-characterized miRNAs and find that the vast majority of their effects are indeed modest in magnitude. However, this method is useful in studying the downstream effects of microRNAs. The major limitation of proteomics remains the low number of detected proteins and the inability to reliably detect modest effects. Furthermore, since many miRNAs show only 30–60% reduction of target expression in Reporter-UTR assays, prevalent thresholds (like fold change greater than 2) in proteomics studies are not suitable for miRNA-proteomics studies.

## Materials and Methods

Constructs: hsa-miR-34a was expressed from pSilencerTM 4.1-CMV neo vector (Ambion). The construction of clone and design of complementary lock nucleic acid (LNA)-modified oligonucleotides against miRNA has been described in [52]. hsa-miR-29a was expressed from pEGFP-N3 (Clontech). The construction of clone and design of LNA-modified oligonucleotides is described in [53]. We have earlier shown that under these conditions the miRNA is 2.5 fold over-expressed in case of hsa-miR-34a [52] and 14 fold over-expressed in case of hsa-miR-29a [53]. For the generation of luciferase reporter constructs 614 bp, 292 bp and 378 bp fragments of VDAC1, VDAC2 and VDAC3 respectively were amplified using cDNA from HEK293 T cell line (primers VDAC 1–3′UTR F and R, VDAC 2–3′UTR F and R and VDAC 3–3′UTR F and R, Figure S2). The fragments were cloned into multiple cloning sites (Xho I and Not I) of the pSicheck-2 dual luciferase reporter plasmid (Promega). Each of the clones were confirmed by sequencing.

Real time analysis: The miRNA expression was quantified in real-time using TaqMan® miRNA assays for both miRNA-29a and 34a according to the manufacturer’s directions (Applied Biosystems Inc., Foster City, CA).hsa-miR-92a was used as endogenous control for expression analysis. Briefly, reverse transcriptase (RT) reactions were performed with miRNA-specific RT primers and 2 ug of total RNA for 30 min at 37°C followed by 10 min incubation at 95°C to inactivate the RT enzyme. End-point PCR was then performed using the RT product and miRNA-specific PCR primers for 40 cycles.

Cell culture and SILAC: Human embryonic kidney cell line HEK293T (ATCC number CRL-11268) was cultured in high glucose Dulbecco’s modified eagle’s medium (DMEM, Invitrogen) supplemented with dialyzed fetal bovine serum. For preparing SILAC medium, SILAC™ Protein ID and Quantitation Media Kit (MS10030) from Invitrogen was used. A pool of cells was grown in medium containing ^13^C_6_ Arginine and ^13^C_6_ Lysine (Heavy medium) for 14 days. Complete incorporation of heavy isotope was confirmed using Mass spectrometry. Simultaneously cells were also grown in normal DMEM (Light medium), to maintain similar passage number. In case of hsa-miR-34a, the light cells were transfected with antisense LNA-modified oligonucleotides (40 nM). The heavy cells were transfected with pSilencer construct expressing hsa-miR-34a. In case of hsa-miR-29a, heavy cells were transfected with antisense LNA-modified oligonucleotides (40 nM), whereas light cells were transfected with pEGFP-N3 construct expressing hsa-miR-29a. All transfections were performed in T25 culture flasks using lipofectamine (Invitrogen) as per manufacture’s protocol. The cells were harvested 24 hours after transfection in case of miR-29a and 36 hours post transfection in case of miR-34a.

Mass spectrometry: Harvested cells were then washed thrice with ice cold PBS. RIPA buffer (50 mM Tris-Cl pH 7.4, 150 mM NaCl, 2 mM EDTA, 2% NP40 and 1X Protease Inhibitor) was used to prepare cell lysate. Debris was removed by centrifugation. The protein was quantified using BCA method. Hundred microgram of each cell lysate was pooled for miR-34a and miR-29a separately and used for Methanol-Chloroform precipitation.

MudPIT: The protein pellet for miR-34a was dissolved in solution containing 0.2% ProteasMAX (Promega) and 4 M Urea in 50 mM Ammonium bicarbonate. The protein was reduced (5 mM tris(2-carboxyethyl)phosphine), alkylated (10 mM iodoacetamide) and trypsinized (1 microgram trypsin : 25 microgram protein) (Promega). Formic acid was added to final concentration of 5% of the peptide pool. The mixture was spun to remove the debris. The peptides were loaded on 250-µm i.d. fused capillary column, packed with 2.5 cm long 5 µm SCX (Luna, Phenomenex) and 2.5 cm of 10 µm Jupiter Reverse phase resin (Phenomenex). The peptides were desalted using this set-up. The SCX end of the this column was now attached to 100 µm i.d. fused capillary with a pulled tip and packed with 15 cm of 4 µm Jupiter Reverse phase resin (Phenomenex). This was the connected inline with Eksigent pump (Eksigent Technologies). The buffer solution that were used are: 5% acetonitrile with 0.1% formic acid (buffer A); 80% acetonitrile with 0.1% formic acid (buffer B), and 500 mM ammonium acetate with 5% acetonitrile and 0.1% formic acid (buffer C). For each sample seven steps chromatography was performed. For first step 70 minute gradient was used with 0 to 3 min –100% buffer A, 3 to 10 min of 0–15% buffer B, 10 to 60 min of 15–45% buffer B and 60 to 70 min of 45–100% buffer B and then back to 100% buffer A. for step 2 to 7, 180 minutes of gradient was used where 0 to 3 min –100% buffer A, 3 to 8 min of salt bump (buffer C), 8 to 20 min of 0–15% buffer B, 20 to 150 min of 15–45% buffer B and 150 to 170 min of 45–100% buffer B and then last 10 minutes of 100% buffer A. Six different salt bumps that were used are: 10, 20, 30, 40, 60 and 100%. The eluting peptides were then directly connected to hybrid LTQ linear ion trap-Orbitrap (Thermo Fisher). The data was acquired in data dependent mode with a full scan between 400–2000 m/z at 60000 resolution in the Orbitrap. Full scan was succeeded by five ms2 scans for topmost peaks in linear ion trap with normalized collision energy of 35%. The parameters that were used for dynamic exclusion are as follows: repeat count - 2, repeat duration –30, list size –50, exclusion duration –60.

Gel based method: The pellet for miR-29a was dissolved in RIPA buffer with 0.1% SDS, which was loaded on 5–12% Bis-Tris gel. The gel was stained with Colloidal commassie blue solution. Sample lane was then cut from rest of the gel and sliced into ten pieces. Individual piece was then cut into smaller approximately one mm^3^ size and washed in 50% acetonitrile to destain the pieces. The gel pieces were then dehydrated, reduced (100 mM DTT), alkylated (50 mM iodoacetamide) and trypsinized (1 microgram trypsin: 25 microgram protein) (Promega). The peptides were extracted by washing with 50% and 100% acetonitrile. The peptides were then vacuum dried and resuspended in 100 mM ammonium bicarbonate with 5% formic acid. The peptides were then loaded in split free nano- LC system (EASY-nLC; Proxeon Biosystems now Thermo Fisher Scientific) autosampler. The buffer solutions used for chromatography are: 2% acetonitrile with 0.1% formic acid (buffer A); 100% acetonitrile with 0.1% formic acid (buffer B). A gradient of 120 minutes was then used each fraction. The gradient was 0 to 108 min of 3–35% Buffer B, 108 to 110 min of 35–100% buffer B and last ten minutes of buffer B. The eluent peptides were coupled to hybrid LTQ linear ion trap-Orbitrap (ThermoFisher). The full scans were performed between 350–2000 m/z at 60000 resolution in Orbitrap. Each full scan was then followed by six data dependent ms2 scans in ion trap with normalized collision energy of 35%. The parameters that were used for dynamic exclusion are as follows: repeat count −1, repeat duration –20, list size –500, exclusion duration –40.

Mass spectrometry data analysis: Protein and phosphopeptide identification, quantification and phospho analysis were done with Integrated Proteomics Pipeline - IP2 (Integrated Proteomics Applications, Inc., San Diego, CA. http://www.integratedproteomics.com/) using ProLuCID, DTASelect2, Census, DeBunker and Ascore. The acquired raw files were converted into ms1 and ms2 files using Rawextract 1.9.5 [54]. The data was searched with the ProLuCID [55] algorithm against EBI IPI Human protein database version 3.44 (http://www.ebi.ac.uk/IPI/IPIhuman.html, released on May 20, 2008), concatenated with decoy database. In order to accurately estimate peptide probabilities and false discovery rates, we used a decoy database containing the reversed sequences of all the proteins appended to the target database [56]. Tandem mass spectra were matched to sequences using the ProLuCID [55]algorithm with 4.5 amu peptide mass tolerance. The search space included all fully-, half- and non-tryptic peptide candidates that fell within the mass tolerance window with no miscleavage constraint. Search parameters includes: Static modifications: carbamidomethylation at cysteine (57.02146 amu) and Heavy modification at lysine and arginine (6.0204 amu) for heavy search; dynamic modifications: oxidation of methionine (15.9949 amu) and deamidation of asparagine and glutamine (0.984 amu) for both light and heavy search. ProLuCID searches were done on an Intel Xeon cluster running under the Linux operating system. The validity of peptide spectrum matches (PSMs) was assessed in DTASelect [57], [58] using two SEQUEST [59] defined parameters, the cross-correlation score (XCorr), and normalized difference in cross-correlation scores (DeltaCN). The search results were grouped by charge state (+1, +2, +3, and greater than +3) and tryptic status (fully tryptic, half-tryptic, and non-tryptic), resulting in 12 distinct sub-groups. In each one of these sub-groups, the distribution of Xcorr, DeltaCN, and DeltaMass values for (a) direct and (b) decoy database PSMs was obtained, then the direct and decoy subsets were separated by discriminant analysis. Full separation of the direct and decoy PSM subsets is not generally possible; therefore, peptide match probabilities were calculated based on a nonparametric fit of the direct and decoy score distributions. A peptide confidence threshold was dynamically set and only peptides with delta mass less than 10 ppm were accepted to achieve protein level false discovery rate below 1%. After this last filtering step, we estimate that the protein and peptide false discovery rates were below 1% and 0.25%, respectively.

This data was then used as input for quantitative analysis performed using Census [60], [61]. The data was corrected if the mode of ratios was not zero. Arginine to proline conversion was corrected in Census. Pair-wise Student’s t test was performed based the Census Light/Heavy protein abundance ratio of the replicates of mir-29 and mir-34 using two-tail, assuming unequal variance, for each protein. For each protein, a p value for the null hypothesis of no differential expression (i.e. log2 ratio  = 0) was derived from the means of the light and heavy protein ratios. Proteins were considered to be significantly changed based on a p-value threshold of 0.05, which was corrected for multiple testing by the procedure of Benjamini-Hochberg [62]. The quantified proteins were used for further analysis. In case of miR-34a, reciprocal of the ratios was estimated so as to be compared directly to miR-29a. Raw data files and data analysis parameter files can be found at http://fields.scripps.edu/published/miR-34a_miR-29a_Proteomics_2012/.

miRNA – target prediction and functional classification: The prediction data for hsa-miR-29a and hsa-miR-34a was retrieved from http://mirecords.biolead.org/ [63]. In total there are ∼31,000 and 33000 predicted targets for hsa-miR-29a and hsa-miR-34a respectively. We used filtering criteria where at least four other prediction programs and Target Scan should predict a gene as target to derive a list of consensus list of targets for miRNAs. The Refseq ids were converted to IPI ids using an online server (http://biit.cs.ut.ee/gprofiler/gconvert.cgi) [64], to get a list of predicted list of potential targets. Gene Ontology classification was done using DAVID [65], [66].

Luciferase assay: For reporter gene assays HEK 293T cells were seeded at 20,000 cells per 96-well dish and transfected 24 hours later using Lipofectamine 2000 reagent (Invitrogen, USA) as described by the manufacturer. The cells were at 70% confluency at the time of transfection. Each co transfection reaction contained 200 ng of DNA comprising of pEGFPN3 expressing mir-29a and pSicheck 2 containing 3′UTR in 1∶1 ratio which was normalized to a transfection set containing pEGFPN3 (miRNA expression vector control) and pSicheck 2 containing 3′UTR in 1∶1 ratio. After 24 hours, cells were lysed and luciferase activity was analyzed in dual-luciferase assay (Promega, USA) using Top count luminometer NXT (Perkin Elmer).Renilla luciferase activity was normalized to Firefly luciferase activity. In all the experiments, transfection and luciferase assays were performed in triplicates.

Western blotting: 30 µg of protein for VDAC1 was resolved on 15% SDS PAGE and electro blotted onto a nitrocellulose membrane using TE 77 semidry transfer unit, Amersham Biosciences at 135 mA for 2 hours. Antibody for VDAC1 (ab15895) (1∶500) was from Abcam. The antibody for GAPDH (sc-32233) (1∶1000) was from sigma. The western blots were imaged on the Odyssey Imager (Licor Biosciences) and band intentisites were quantified using methodology recommended by manufacturer. Band Intensities of GAPDH were used to normalize for equal loading in lanes.

## Supporting Information

Figure S1 **Differential expression of miR-29a and miR-34a.** HEK293T cells were transfected with mock LNA, LNA modified anti-miR-29a (A), Vector or plasmid over-expressing miR-29a (B). RT-PCR was performed using Taqman probes for miR-29a as per manufacturer’s protocols. (C, D) HEK293T cells were transfected with mock LNA, LNA modified anti-miR-34a (C), Vector or plasmid over-expressing miR-34a (D). Experiments for miR-34a were done similarly. Error bars represent standard error of three replicates (n = 3).
(TIF)

Figure S2 **Primer Sequences for cloning of 3′UTR of VDAC.**
(TIF)

Table S1 **Entire list of proteins obtained in miR-34a proteomics study.**
(XLS)

Table S2 **Entire list of proteins obtained in miR-29a proteomics study.**
(XLS)

Table S3 **List of targets of miR-34a obtained from consensus target prediction.**
(XLS)

Table S4 **List of targets of miR-29a obtained from consensus target prediction.**
(XLS)
