# Supplementary figures and images for: Identification of Novel Targets for miR-29a Using miRNA Proteomics
Source: PLoS One. 2012 Aug 27;7(8):e43243. doi: 10.1371/journal.pone.0043243 (PMC3428309; doi:10.1371/journal.pone.0043243)

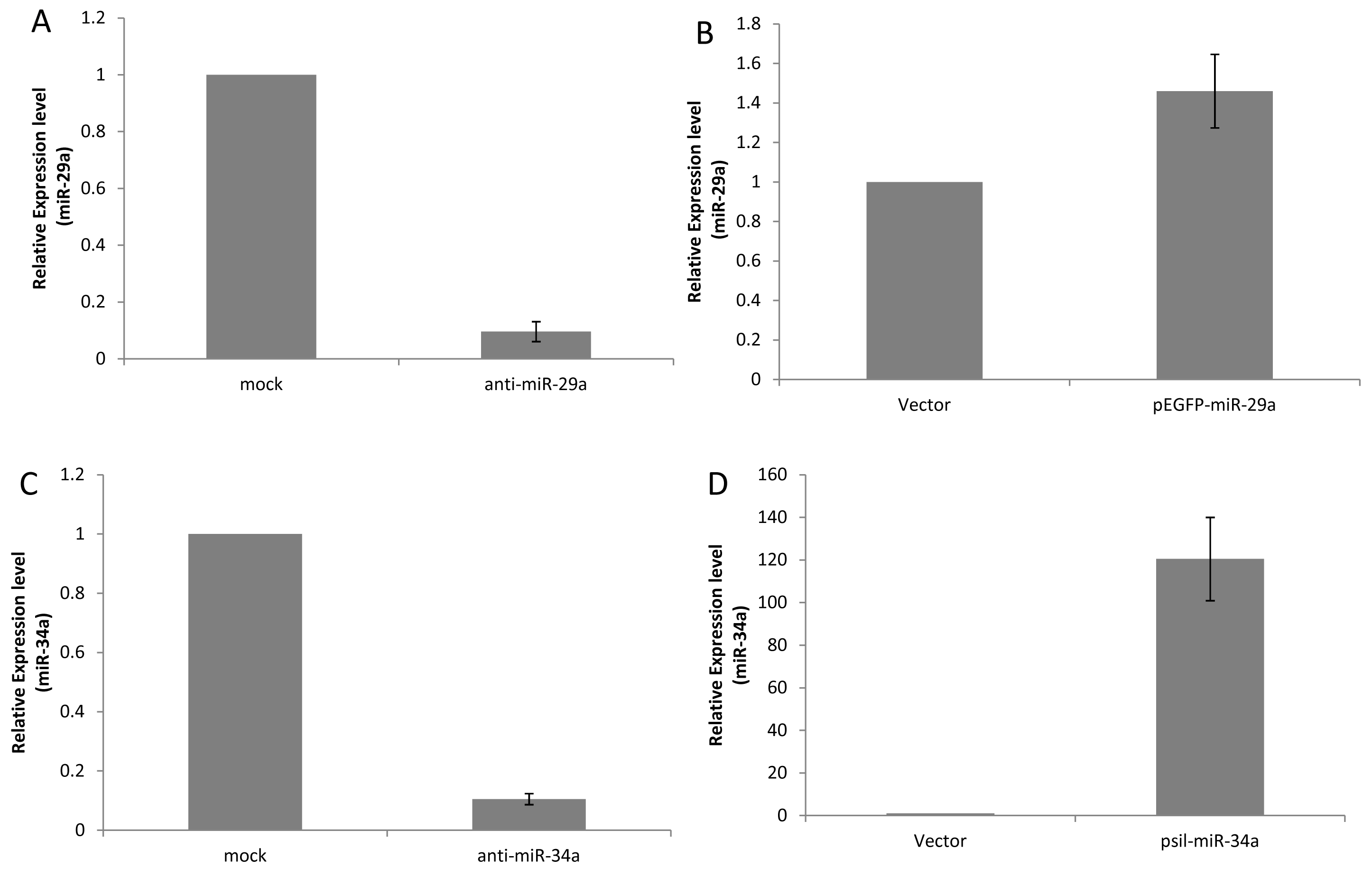

Supplement: Figure S1 — Differential expression of miR-29a and miR-34a. HEK293T cells were transfected with mock LNA, LNA modified anti-miR-29a (A), Vector or plasmid over-expressing miR-29a (B). RT-PCR was performed using Taqman probes for miR-29a as per manufacturer’s protocols. (C, D) HEK293T cells were transfected with mock LNA, LNA modified anti-miR-34a (C), Vector or plasmid over-expressing miR-34a (D). Experiments for miR-34a were done similarly. Error bars represent standard error of three replicates (n = 3). (TIF) [file pone.0043243.s001.tif]

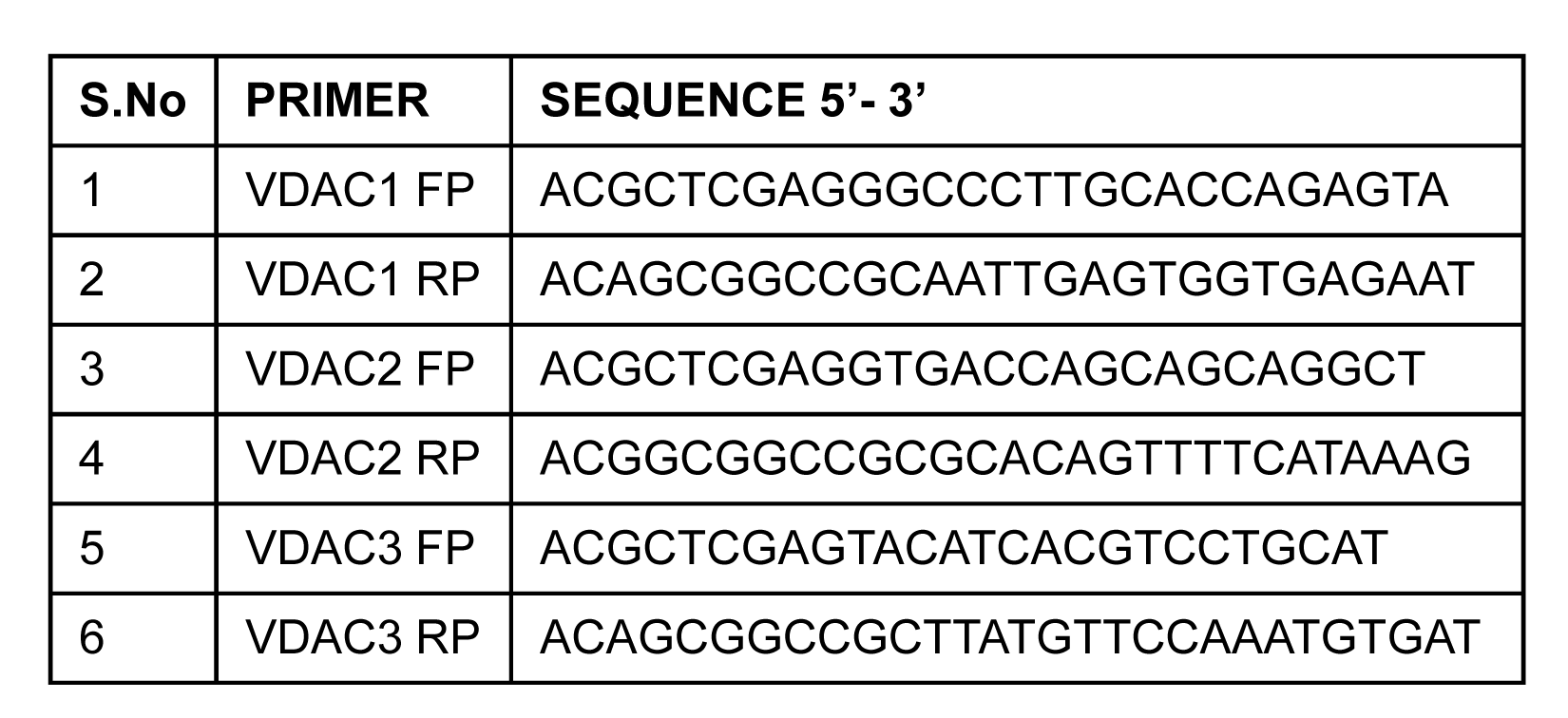

Supplement: Figure S2 — Primer Sequences for cloning of 3′UTR of VDAC. (TIF) [file pone.0043243.s002.tif]
